# A Bicyclic Diterpenoid with a New 15,16-Dinorlabdane Carbon Skeleton from *Leonurus japonicus* and Its Coagulant Bioactivity

**DOI:** 10.3390/molecules181113904

**Published:** 2013-11-11

**Authors:** Fu Peng, Liang Xiong, Xiao-Mei Zhao

**Affiliations:** 1 State Key Laboratory Breeding Base of Systematic Research, Development and Utilization of Chinese Medicine Resources, Sichuan Province and Ministry of Science and Technology, Chengdu 610075, China; E-Mail: fujing126@yeah.net; 2 School of Life Sciences, Sichuan University, Chengdu 610064, China; 3 Pharmacy College, Chengdu University of Traditional Chinese Medicine, Chengdu 610075, China; E-Mail: xiaomei08gz@163.com

**Keywords:** *Leonurus japonicus*, dinorlabdane diterpenoid, coagulant activity

## Abstract

A new 15,16-dinorlabdane diterpenoid **1** and a known labdane diterpenoid **2**, together with three known ergosterols **3**–**5**, were isolated from the EtOAc-soluble portion of the EtOH extract of *Leonurus japonicus*. Their structures were elucidated by physical and spectroscopic analysis. Compound **1** showed *in vitro* coagulant activity in the APTT, PT, TT, and FIB assays.

## 1. Introduction

**Figure 1:**
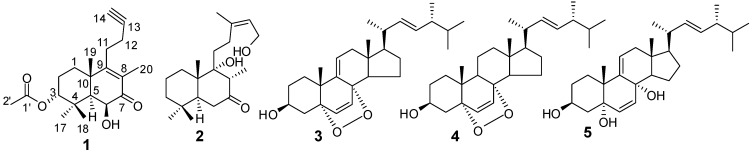
Structures of compounds **1**–**5**.

*Leonurus* *japonicus* Houtt. (Lamiaceae) is an annual or biennial herbaceous plant widely distributed and cultivated in China. The dried herb is used in Traditional Chinese Medicine for the treatment of various diseases, especially menstrual disturbances, dysmenorrhea, and amenorrhea [1]. Our previous investigation on the plant resulted in the isolation of 19 secondary metabolites, including sesquiterpenoids, coumarins, lignans and phenylpropanoids [2,3]. In continuation of our research on bioactive natural products from *L. japonicus*, we have now carried out another study on the same EtOH extract. The present study has led to the isolation of diterpenoids and ergosterols, including a new norditerpenoid, (−)-3*α*-acetoxy-6*β*-hydroxy-15,16-dinorlabd-8(9)-ene-13-yne-7-one (**1**), and four known compounds, leoheteronin F (**2**) [4], (22*E*,24*R*)-5*α*,8*α*-epidioxyergosta-6,9(11),22-trien-3*β*-ol (**3**) [5], (22*E*,24*R*)-5*α*,8*α*-epidioxyergosta-6,22-dien-3*β*-ol (**4**) [5], and (22*E*)-ergosta-6,9,22-triene-3*β*,5*α*,8*α*-triol (**5**) [6] (Figure 1). Compound **1**, an unusual bicyclic diterpenoid possessing a new 15,16-dinorlabd carbon skeleton, showed coagulant activity by shortening the activated partial thromboplastin time (APTT), prothrombin time (PT), and thrombin time (TT), and increasing the fibrinogen (FIB) levels. This paper reports the isolation, structure elucidation, and bioassays of these compounds.

## 2. Results and Discussion

Compound **1** was obtained as a colorless oil, and its IR spectrum showed the presence of OH (3,456 cm^−1^), acetylenic bond (3,291 and 2,120 cm^−1^), carbonyl (1,728 and 1,659 cm^−1^) and double bond (1,607 cm^−1^) groups. The molecular formula C_20_H_28_O_4_ of **1**, with seven degrees of unsaturation, was indicated by the HRESIMS peak at *m*/*z* 355.1883 [M+Na]^+^ (calcd. for C_20_H_28_O_4_Na, 355.1885). The ^1^H-NMR spectrum of **1** displayed resonances attributable to five tertiary methyl groups [*δ*_H_ 1.00 (H_3_-17), 1.42 (H_3_-18), 1.48 (H_3_-19), 1.81 (H_3_-20), and 1.99 (H_3_-2ʹ)], a monosubstituted acetylenic bond [*δ*_H_ 2.47 (t, *J* = 2.4 Hz, H-14)], two oxymethines [*δ*_H_ 4.60 (t, *J* = 3.0 Hz, H-3), 4.19 (dd, *J* = 4.2, 3.0 Hz, H-6)], an aliphatic methine [*δ*_H_ 1.97 (d, *J* = 3.0 Hz, H-5)], and four aliphatic methylenes between *δ*_H_ 1.70 and 2.60 (Table 1). It also displayed an exchangeable resonance assignable to a secondary hydroxy proton at *δ*_H_ 4.53 (d, *J* = 4.2 Hz, OH-6). The ^13^C-NMR and DEPT spectra of **1** showed a total of 20 carbon resonances corresponding to the above protonated units and six quaternary carbons, including a conjugated carbonyl (*δ*_C_ 198.1) and a conjugated tetrasubstituted double bond (*δ*_C_ 166.5 and 129.6) which implied an *α*,*β*-unsaturated ketone system for compound **1**. The presence of an acetoxyl group in **1** was indicated by the signals of the ester carbonyl (*δ*_C_ 170.4) in the ^13^C-NMR and the methyl (*δ*_H_ 1.99) in the ^1^H-NMR. In addition, the signals of the sp quaternary carbon (*δ*_C_ 83.9) and sp methine (*δ*_C_ 70.6, *δ*_H_ 2.47) were characteristic of a terminal alkynyl group. These spectroscopic data suggested that **1** was an unusual bicyclic dinorditerpenoid possessing an *α*,*β*-unsaturated ketone system, a terminal alkynyl unit, and substitutions of a hydroxyl and an acetoxyl group.

**Figure 2:**
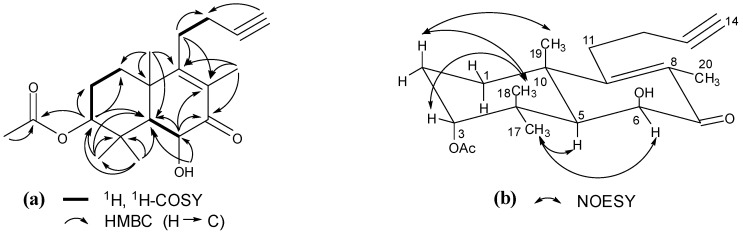
(**a**) Key ^1^H, ^1^H-COSY and HMBC correlations of **1**; (**b**) Key NOESY correlations of **1**.

The above conjecture was confirmed by 2D NMR data analysis. The proton and corresponding carbon resonances in the 2D NMR spectra of **1** were assigned by the gHSQC experiment. The H_2_-1/H_2_-2/H-3, H-5/H-6, and H_2_-11/H_2_-12 coupling correlations in the gCOSY spectrum revealed three vicinal coupled systems (Figure 2). In the HMBC spectrum, two- and three-bond correlations of H-3/C-1, C-2, C-4, C-5, C-17, C-18, and C-1ʹ; H-6/C-4, C-5, C-7, C-8, and C-10; OH-6/C-5 and C-6; H_2_-11/C-8, C-9, C-10, C-12, and C-13; H-14/C-12; H_3_-17 and H_3_-18/C-3, C-4, and C-5; H_3_-19/C-1, C-5, C-9, and C-10; H_3_-20/C-7, C-8, and C-9 (Figure 2), in combination with the vicinal coupled signals, indicated that the planar structure of **1** was 3-acetoxy-6-hydroxy-15,16-dinorlabd-8(9)-ene-13-yne-7-one.

**Table 1:** ^1^H- (600 MHz) and ^13^C-NMR (150 MHz) data of **1** (in Me_2_CO-*d*_6_, *δ* in ppm, *J* in Hz).

No.	*δ*_H_	*δ*_C_	No.	*δ*_H_	*δ*_C_
1	1.73 m	31.7	12	2.37 m	18.5
2	2.16 m, 1.72 m	23.5	13	–	83.9
3	4.60 t (3.0)	78.5	14	2.47 t (2.4)	70.6
4	–	38.0	17	1.00 s	27.5
5	1.97 d (3.0)	48.5	18	1.42 s	24.4
6	4.19 dd (4.2, 3.0)	71.4	19	1.48 s	22.1
7	–	198.1	20	1.81 s	11.9
8	–	129.6	1ʹ	–	170.4
9	–	166.5	2ʹ	1.99 s	21.0
10	–	41.5	OH-6	4.53 d (4.2)	–
11	2.58 t (7.8)	29.5			

The configuration of **1** was elucidated on the basis of the NOESY interactions and interproton coupling patterns [7,8,9]. A relatively small coupling constant of H-3 (*J* = 3.0 Hz) and the distinct NOE between H-3 and H_3_-18 (Figure 2) confirmed the assignment of H-3 as *β*-equatorial orientation [8]. The H-5 and H-6 coupling constant (*J* = 3.0 Hz) placed the two protons *cis* to each other in an *α*-axial, *α*-equatorial orientation, leaving the OH-6 as *β*-axial, which was confirmed by the NOE correlations of H_3_-17 with H-5 and H-6. These data conclusively proved the new structure of **1** as (−)-3*α*-acetoxy-6*β*-hydroxy-15,16-dinorlabd-8(9)-ene-13-yne-7-one.

The coagulant activity of the compounds was examined by monitoring activated partial thromboplastin time (APTT), prothrombin time (PT), thrombin time (TT), and fibrinogen (FIB) levels. A summary of the results for compound **1** is shown in Table 2. At concentrations of 1 mg/mL and 0.1 mg/mL, compound **1** shortened the APTT, PT, and TT and increased the content of FIB. Compared to the normal saline control group, they were statistically significant (*p* < 0.05 or *p* < 0.01), except for the PT at 0.1 mg/mL. Although there was no statistical difference in the APTT, PT, and TT between the group of 0.01 mg/mL and the normal saline group, treatment with compound **1** at this concentration resulted in obviously increased FIB (*p* < 0.05). The above results showed that compound **1** had coagulant activity via multiple pathways. Other isolated compounds were inactive in the blood coagulation assay at the concentrations of 1 mg/mL, 0.1 mg/mL, and 0.01 mg/mL.

**Table 2:** Coagulant activity of compound **1** ^a^.

Sample	Dose	APTT (s)	PT (s)	TT (s)	FIB (g/L)
Control	Normal saline	31.22 ± 6.20	9.98 ± 0.54	53.24 ± 5.91	1.59 ± 0.08
Compound 1	1 mg/mL	28.88 ± 5.57 **	9.87 ± 0.47*	49.94 ± 3.46 *	1.64 ± 0.03 *
	0.1 mg/mL	29.66 ± 4.90 *	9.94 ± 0.44	50.77 ± 4.43 *	1.66 ± 0.03 *
	0.01 mg/mL	31.38 ± 4.87	10.00 ± 0.49	53.28 ± 3.98	1.65 ± 0.06 *

^a^ Each value represents the means ± SEM (n = 10); * *p* < 0.05 as compared to control; ** *p* < 0.01 as compared to control.

## 3. Experimental

### 3.1. General

NMR spectra were recorded on a INOVA-500 or Bruker-AVIIIHD-600 spectrometers. HRESIMS were measured with a Waters Synapt G_2_ HDMS. IR spectra were recorded on a Nicolet 5700 FT-IR microscope instrument (FT-IR microscope transmission). Optical rotations were measured with a Perkin-Elmer 341 plus. Column chromatography was performed with silica gel (200–300 mesh, Yantai Institute of Chemical Technology, Yantai, China), MCI gel CHP 20P (75–150 μm, Mitsubishi Chemical, Co., Tokyo, Japan), and Sephadex LH-20 (Amersham Pharmacia Biotech AB, Uppsala, Sweden). HPLC separation was performed on an instrument consisting of a Cometro 6000LDS pump and a Cometro 6000PVW UV/VIS detector with an Ultimate (250 × 10 mm) preparative column packed with C_18_ (5 μm). TLC was carried out with glass precoated silica gel GF_254_ plates (Qingdao Marine Chemical Inc., Qingdao, China).

### 3.2. Plant Material

The *L. japonicus* herb was collected in May of 2012 from a field in Wenjiang District, Chengdu City, Sichuan Province, China. Plant identity was verified by Prof. Min Li (Chengdu University of TCM, Sichuan, China). A voucher specimen (SYMC-0522) was deposited at the School of Pharmacy, Chengdu University of TCM, China.

### 3.3. Extraction and Isolation

The air-dried herb of *L. japonicus* (20 kg) was extracted with 95% EtOH (3 × 160 L) at room temperature for 3 × 72 h. The ethanolic extract was evaporated under reduced pressure to yield a dark brown residue (1.2 kg). The residue was suspended in H_2_O and then successively partitioned into EtOAc (400 g) and n-BuOH (160 g) fractions. The EtOAc extract was subjected to silica gel CC using a gradient elution of petroleum ether–acetone (100:1–0:1) to afford nineteen fractions (F_1_−F_19_) based on TLC analysis. F_6_ was further separated by MCI with a gradient of increasing MeOH (50%–100%) in water to yield six subfractions F_6-1_−F_6-6_. The successive separation of F_6-4_ with Sephadex LH-20 (petroleum ether−CHCl_3_−MeOH, 5:5:1) and with PTLC (petroleum ether−EtOAc 5:1) yielded **1** (6 mg) and **2** (18 mg). F_6-6_ was purified via silica gel CC over petroleum ether–EtOAc (40:1–5:1) to get F_6-6-1_–F_6-6-4_. Separation of F_6-6-2_ by repeated PTLC (petroleum ether−EtOAc 5:1) and reversed-phase semipreparative HPLC (80% MeCN in H_2_O) yielded **3** (7 mg), **4** (21 mg), and **5** (26 mg).

*(−)-3α-Acetoxy-6β-hydroxy-15,16-dinorlabd-8(9)-ene-13-yne-7-one* (**1**): Colorless oil, 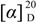 = −17.3 (*c* = 0.10, MeOH); IR νmax: 3456, 3291, 2941, 2874, 2120, 1728, 1659, 1607, 1461, 1375, 1248, 1128, 1047, 930 cm^−1^; ESI-MS *m/z* 355.2 [M+Na]^+^; HRESI-MS: *m/z* 355.1883 [M+Na]^+^ (calcd. for C_20_H_28_O_4_Na, 355.1885); ^1^H- and ^13^C-NMR data see Table 1.

### 3.4. Blood Coagulation Assay [10]

SD rats were lightly anesthetized with ether. Blood was immediately taken from the femoral artery and anticoagulated with 3.8% trisodium citrate (9:1, v/v). Plasma was obtained by centrifugation of the whole blood at 3,500 *g* for 10 min. Then, the plasma (200 μL) was mixed with 20 μL of tested compounds or normal saline and incubated for 5 min at 37 °C. PT, APTT, TT, and FIB were determined by an automatic coagulation analyzer (Sysmex CA-500, Kobe, Japan) according to the manufacturer's instructions. Data were expressed as mean ± SEM (standard error of the mean) of at least three independent experiments. Statistical significance between two groups was determined using the Student’s *t*-test and a *p*-value of <0.05 was considered as significant.

## 4. Conclusions

Plants of the genus *Leonurus* are known to contain terpenoids, especially labdane diterpenoids. Previous phytochemical investigations on *L. japonicus* have led to the isolation of more than 35 labdane diterpenoids [4,9,11,12,13,14,15,16,17]. In the present study, two labdane diterpenoids **1**–**2** and three ergosterols **3**–**5** were obtained from the EtOAc soluble fraction of the EtOH extract of *L. japonicus*. It is worth mentioning that compound **1** is an unusual norditerpenoid with a new 15,16-dinorlabdane carbon skeleton. In addition, ergosterols were isolated from the genus *Leonurus* for the first time. In the *in vitro* assay, compound **1** showed an effect on blood coagulation. The APTT, PT, and TT were shortened and the FIB level was increased simultaneously by compound **1**, which suggested the compound was a potential coagulant.

## Supplementary Materials

Supplementary materials can be accessed at: http://www.mdpi.com/1420-3049/18/11/13904/s1.
