# Supplementary material for: A Bicyclic Diterpenoid with a New 15,16-Dinorlabdane Carbon Skeleton from Leonurus japonicus and Its Coagulant Bioactivity
Source: Molecules. 2013 Nov 11;18(11):13904–9. doi: 10.3390/molecules181113904 (PMC6270439; doi:10.3390/molecules181113904)

Supplemental Materials

List of Contents

No.	Content	Page
1	**Figure S1.** The IR Spectrum of Compound **1**	S2
2	**Figure S2.** The (+)-HRESIMS Spectroscopic Data of Compound **1**	S3
3	**Figure S3.** The ^{1}H-NMR Spectrum of Compound **1** in Me_2CO-d_6	S4
4	**Figure S4.** The ^{13}C-NMR Spectrum of Compound **1** in Me_2CO-d_6	S5
5	**Figure S5.** The DEPT Spectrum of Compound **1** in Me_2CO-d_6	S6
6	**Figure S6.** The gHSQC Spectrum of Compound **1** in Me_2CO-d_6	S7
7	**Figure S7.** The ^{1}H–^{1}H gCOSY Spectrum of Compound **1** in Me_2CO-d_6	S8
8	**Figure S8.** The HMBC Spectrum of Compound **1** in Me_2CO-d_6	S9
9	**Figure S9.** The NOESY Spectrum of Compound **1** in Me_2CO-d_6	S10

Figure S1. The IR Spectrum of Compound **1**.

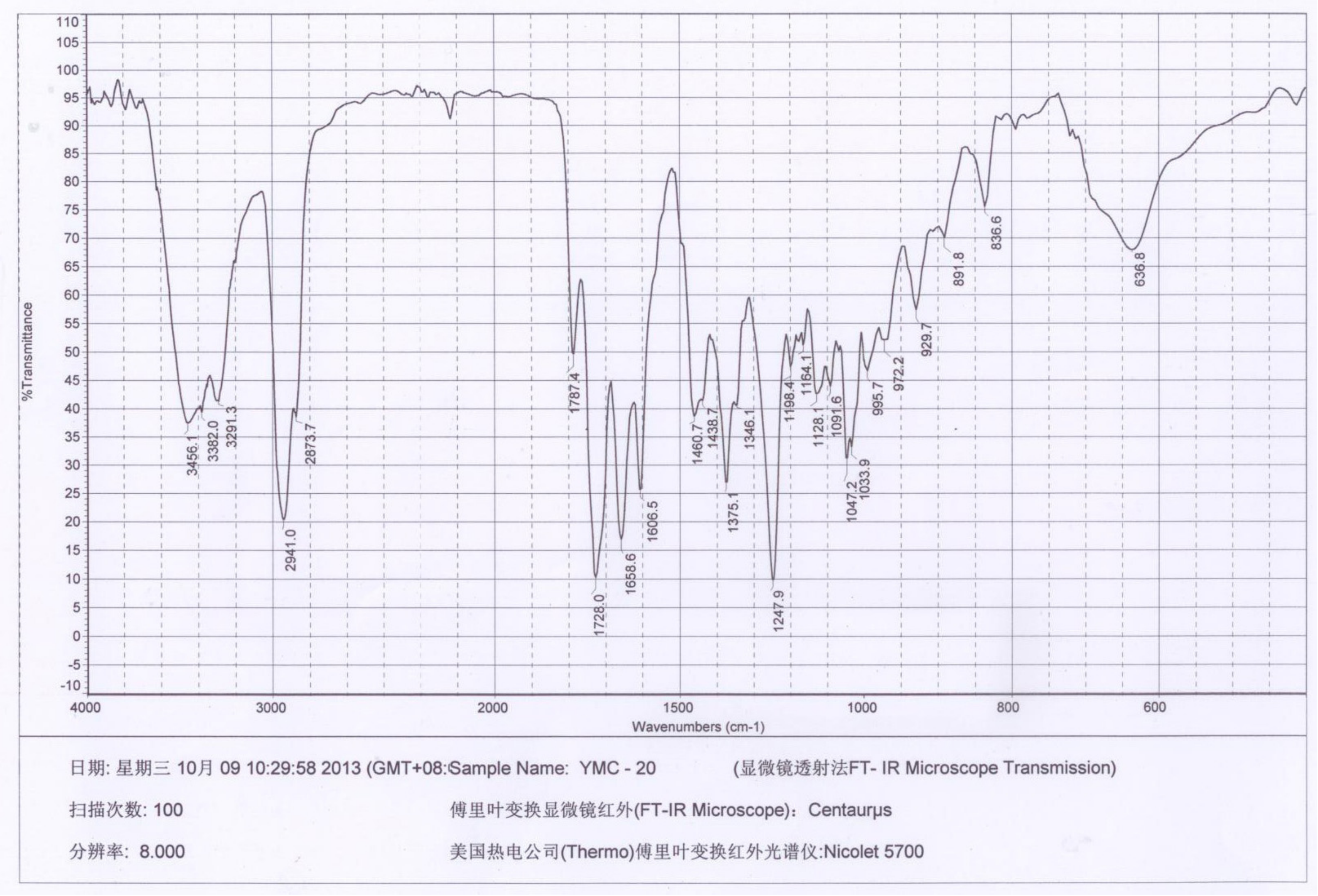

Figure S2. The (+)-HRESIMS Spectroscopic Data of Compound **1.**

Mass	Calc. Mass	mDa	PPM	DBE	Formula	i-FIT	i-FIT Norm	Fit Conf %	C	H	O	Na	K
355.1883	355.1885	-0.2	-0.6	6.5	C20 H28 O4 Na	577.3	0.000	100.00	20	28	4	1	
	355.1887	-0.4	-1.1	1.5	C17 H32 O5 K	595.3	17.987	0.00	17	32	5		1

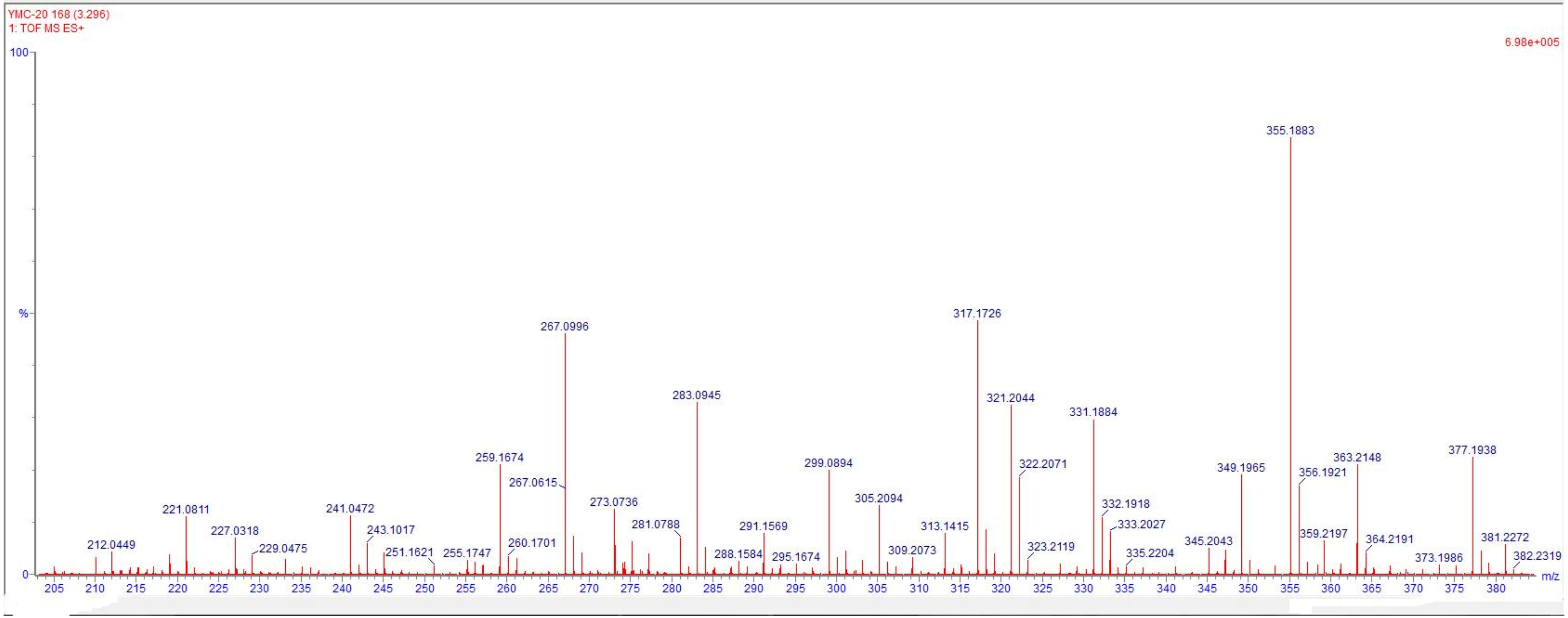

Figure S3. The ^{1}H-NMR Spectrum of Compound **1** in Me_2CO-d_6.

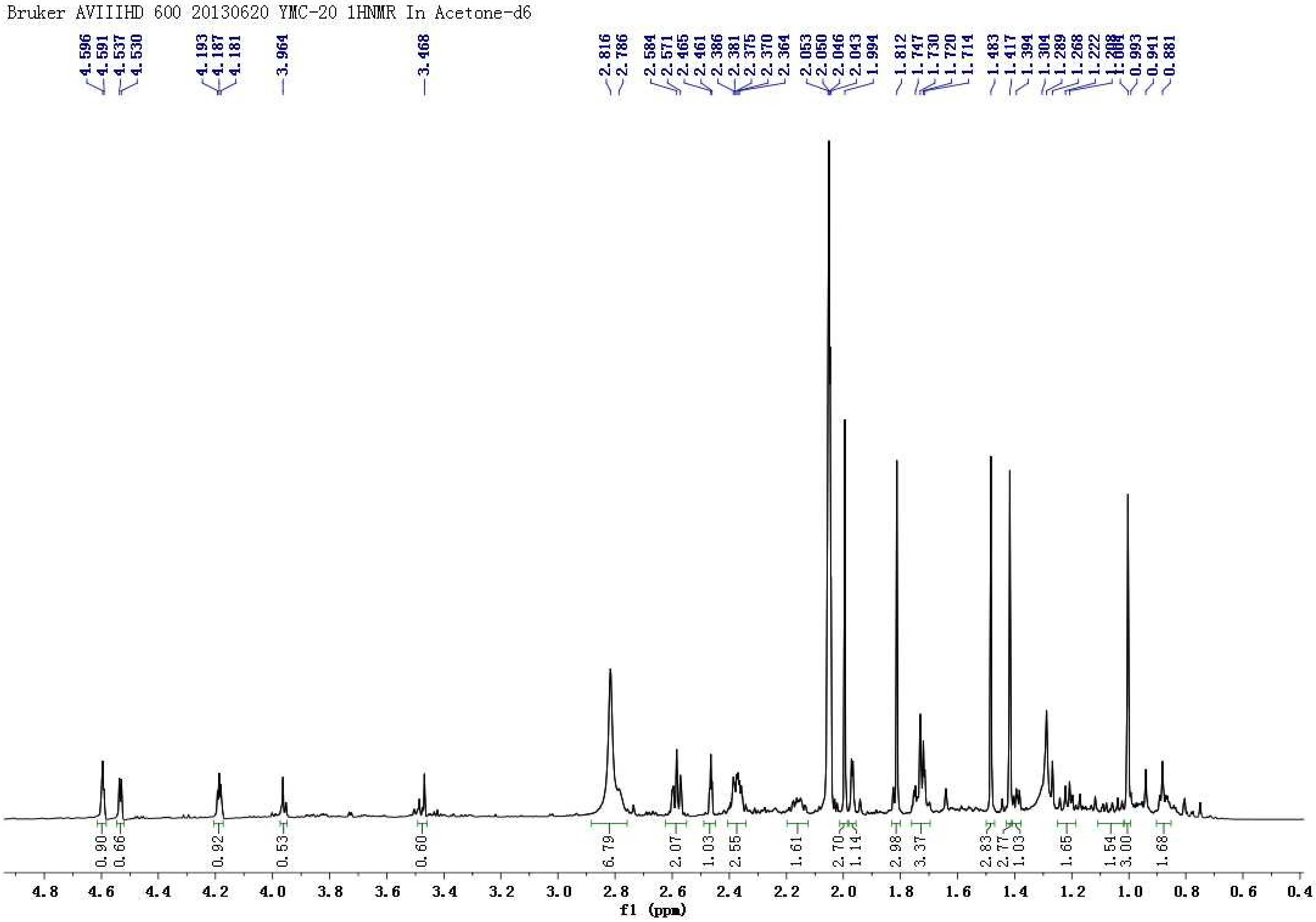

Figure S4. The ^{13}C-NMR Spectrum of Compound **1** in Me_2CO-d_6.

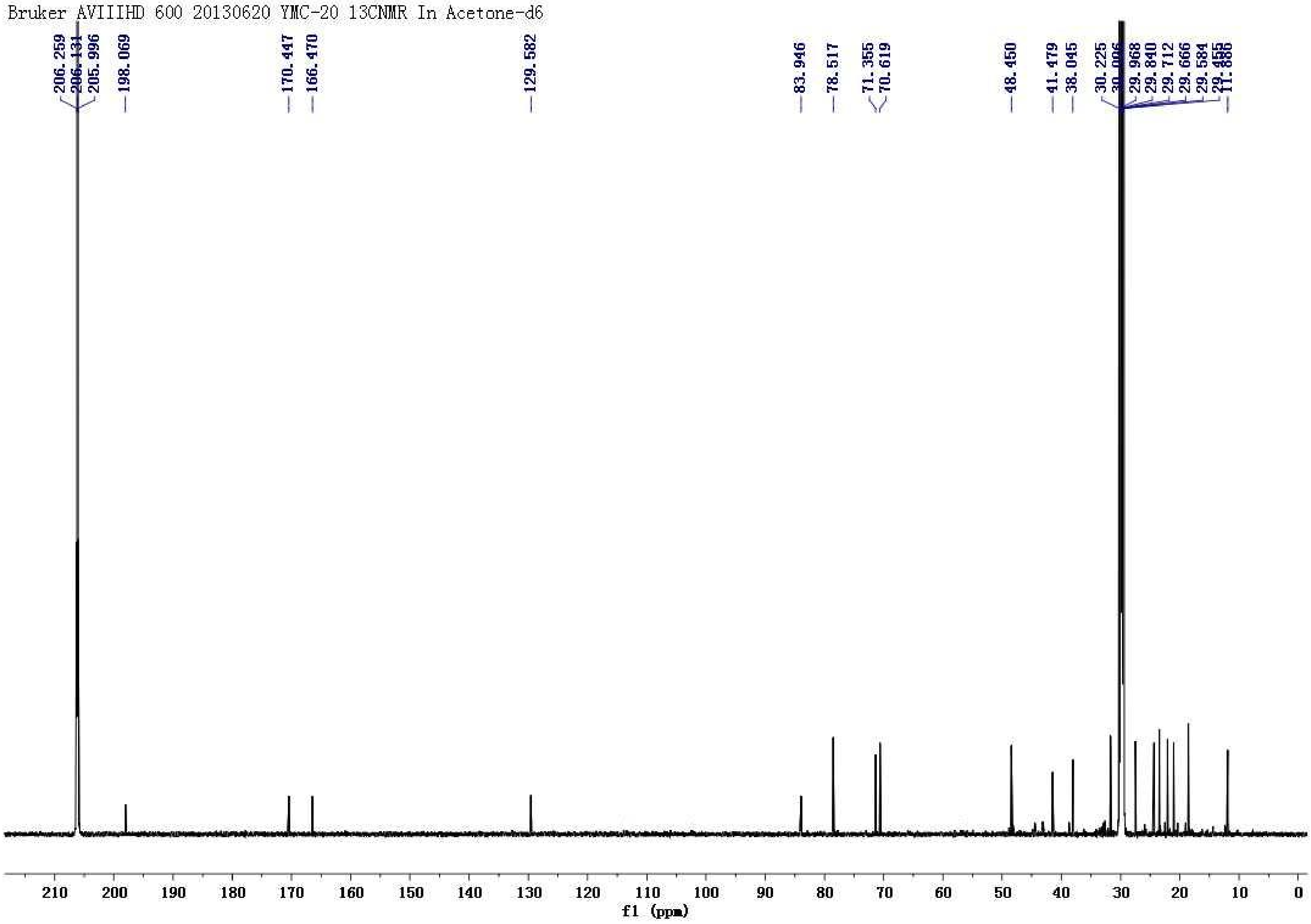

Figure S5. The DEPT Spectrum of Compound **1** in Me_2CO-d_6.

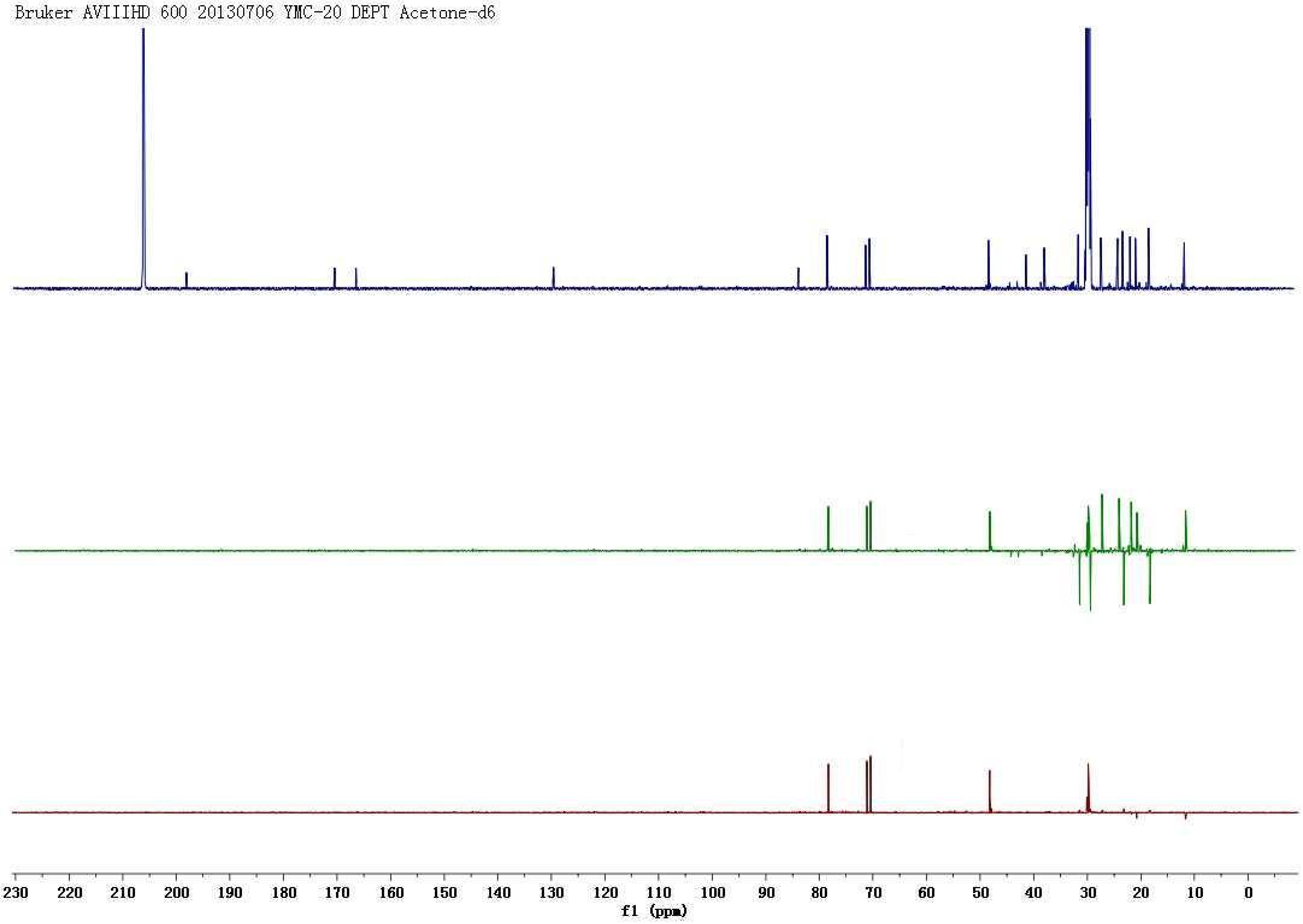

Figure S6. The HSQC Spectrum of Compound **1** in Me_2CO-d_6.

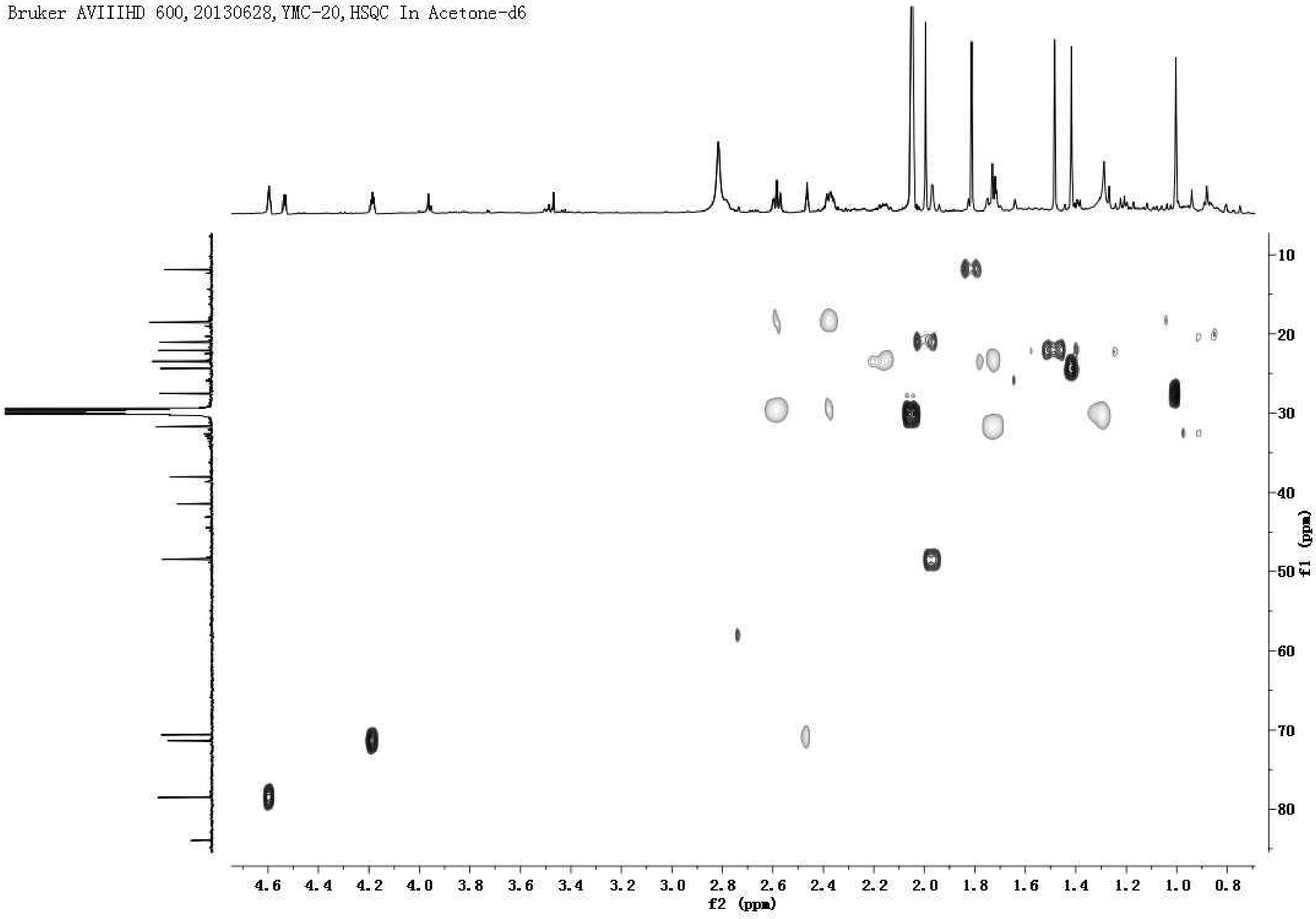

Figure S7. The ¹H–¹H gCOSY Spectrum of Compound **1** in Me_2CO-d_6.

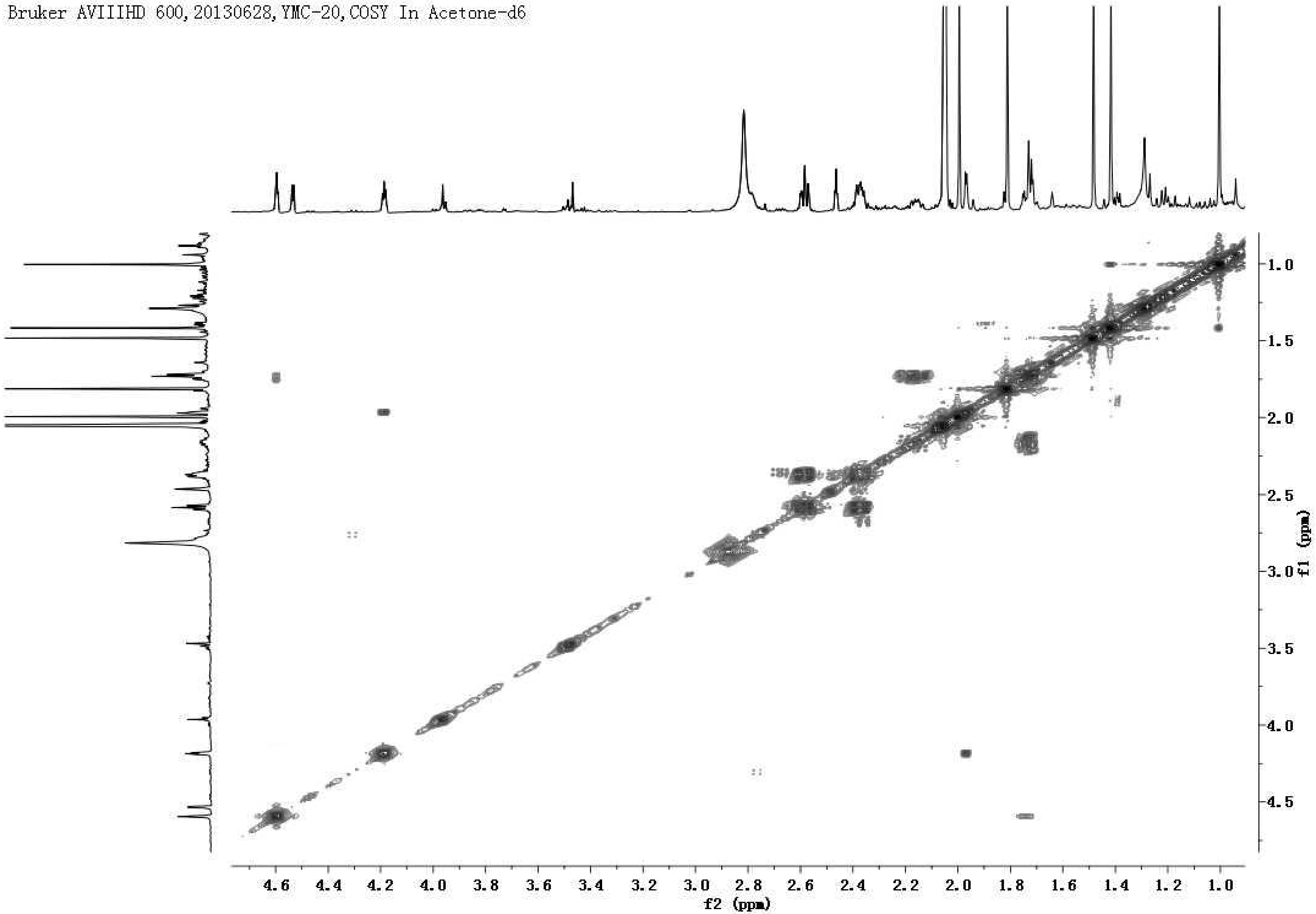

Figure S8. The HMBC Spectrum of Compound **1** in Me_2CO-d_6.

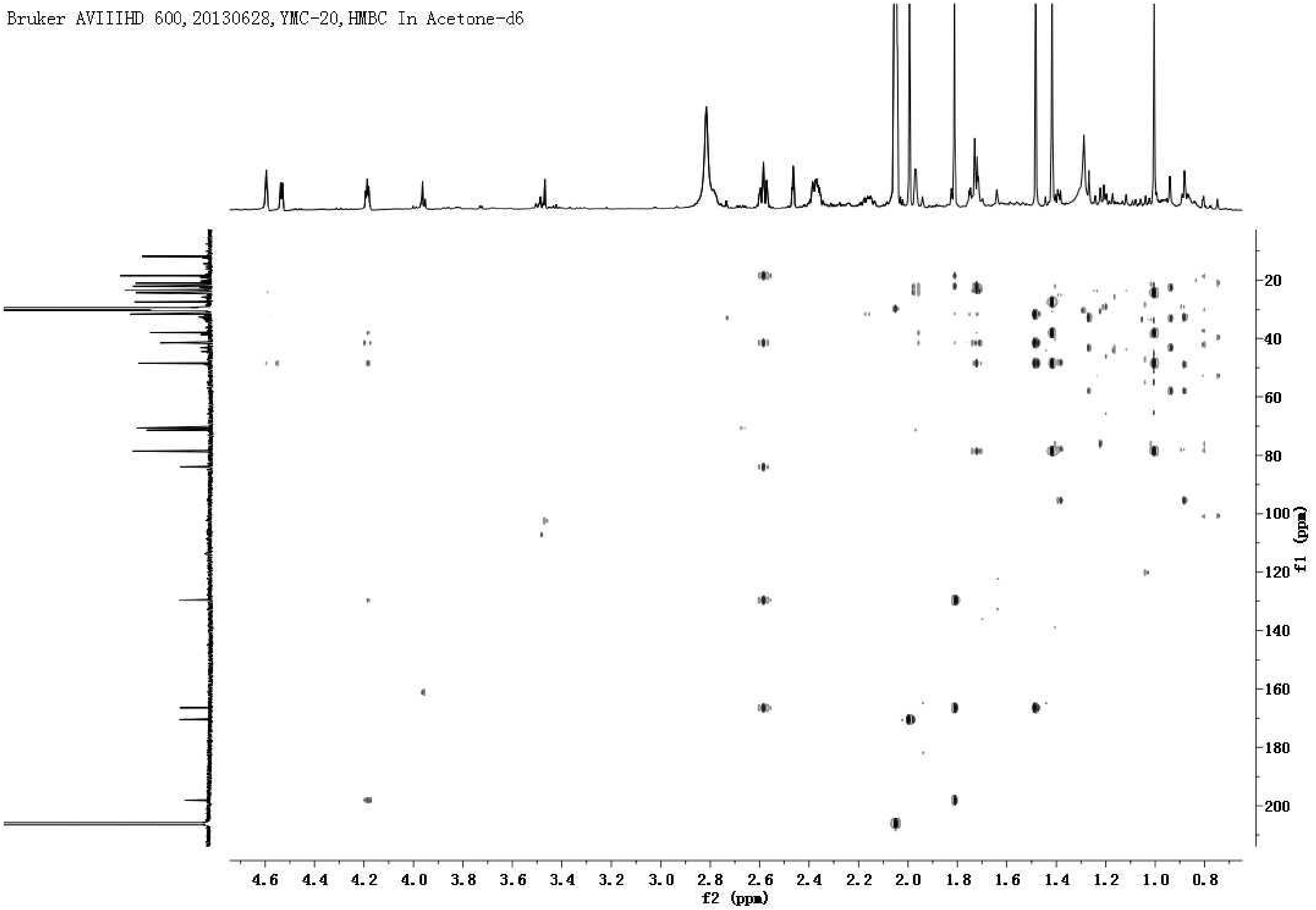

Figure S9. The NOESY Spectrum of Compound **1** in Me_2CO-*d6*.

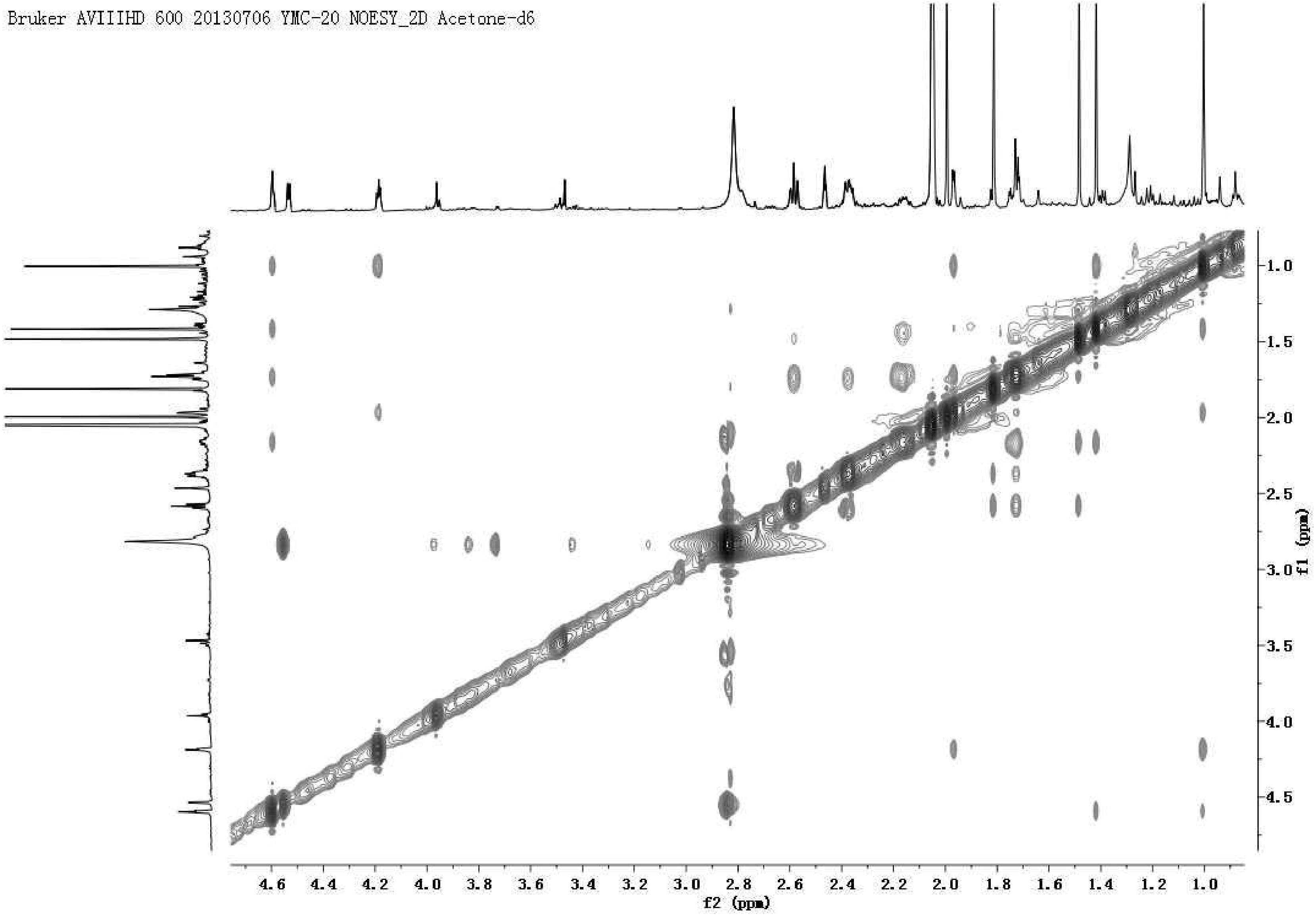

Supplement: Supplementary file 1 [file molecules-18-13904-s001.pdf]
